# Relationship between pharmaceutical pricing strategies with price, availability, and affordability of cardiovascular disease medicines: surveys in Qatar and Lebanon

**DOI:** 10.1186/s12913-019-4828-0

**Published:** 2019-12-18

**Authors:** N. Abdel Rida, M. I. Mohamed Ibrahim, Z. U. D. Babar

**Affiliations:** 1 College of Pharmacy, Qatar University, PO BOX 2713, Doha, Qatar; 2 Department of Pharmacy, University of Huddersfield, Queensgate, Huddersfield, HD1 3DH UK

**Keywords:** Cardiovascular disease, Medicine access, Medicine policy, Generic medicine, Medicines prices

## Abstract

**Background:**

Cardiovascular diseases are the leading cause of death in Lebanon and Qatar. When lifestyle modifications prove insufficient, medication becomes a cornerstone in controlling such diseases and saving lives. Price, availability, and affordability hinder the equitable access to medicines. The study aimed to assess prices, availability, and affordability of essential cardiovascular disease medicines in relation to pricing strategies in Qatar and Lebanon.

**Methods:**

A cross-sectional survey using a variant of the World Health Organization and Health Action International (WHO/HAI) methodology as outlined in “Measuring medicine prices, availability, affordability and price components” (2008), second edition, was adopted. Prices and availability of 27 cardiovascular medicines were collected from public and private dispensing outlets. For international comparison, prices were adjusted to purchasing power parity. Data was analyzed across multiple sectors, within and across countries.

**Results:**

A total of 15 public and private outlets were surveyed in each country. Prices were more uniform in Qatar than in Lebanon. In the public sector, medicines were free-of-charge in Lebanon and priced lower than the international reference prices in Qatar. The ratio of medicine unit price to international reference price in the private sectors surveyed are significantly higher than the acceptable threshold of 4. This ratio of originator brands and lowest priced generics in Qatar were up to two and five times those in Lebanon, respectively, even after adjusting for purchasing power parity. However, prices of lowest priced generics in the private sector were at least 35% cheaper in Qatar and 65% cheaper in Lebanon than their comparative originator brands. Medicines were more available in the private sector in Lebanon than in Qatar, but only the originator brand availability in the public sector in Qatar exceeded the WHO target of more than 80%. While affordable in the public sector in Qatar, four out of thirteen medicines exceeded the threshold in all private sectors covered. Hence, only the public sector in Qatar had a satisfying level of availability and affordability.

**Conclusions:**

Except for the Qatari public sector, medicine prices, availability, and affordability are falling short from targets. Key policy decisions should be implemented to improve access to medicines.

## Background

Medication is a cornerstone in medical disease management and the timely presence and appropriate integration of medication in a treatment plan for short- and long-term ailments are crucial. This is quite evident in the life-long treatment of non-communicable diseases (NCD) such as cardiovascular diseases (CVD) when first-line lifestyle modifications [1–3] are proven inappropriate and unsuccessful. According to the latest WHO global disease burden report (2014), NCD are the leading cause of death worldwide of which CVD accounted for almost half of the death cases. Similar high figures were observed in the Eastern Mediterranean Regional Office of the WHO (EMRO) [4] as well where risk factors for CVD and non-adherence to primary management guidelines are highly prevalent [5–7].

Therefore, ensuring a sustainable access to medicines is deemed indispensable. Access can be defined by the ability of citizens to reach and use pharmaceuticals that are of good quality and affordable, when needed [8]. The price of medicines was one of the barriers to such consistent access [9]. Furthermore, data from few small-scale studies had shown that prices in low-income countries were higher than in richer countries. Several nongovernmental organizations (NGO) and WHO acknowledge that to improve the availability and the affordability of essential medicines, evidence-based national policies and programs must be developed. Sound information based on systematic surveys of standard methodology to evaluate the price and availability of medicines was lacking. As a result, the WHO/HAI Project on Medicine Prices and Availability was established in 2001. After several provisional surveys, the methodology was launched in 2003, and has since been regularly reviewed to increase its efficiency and transparency [9]. Several middle-eastern countries, e.g., Kuwait, Lebanon, and Syria, participated in the first version of WHO/HAI project in 2003, and consequently undertook action towards medicine price reduction and enhancement of availability based on the survey findings and recommendations [10]. A closer look into two middle-eastern countries within the EMRO, Qatar and Lebanon, of different economic indicators demonstrated that cardiovascular diseases are the first leading causes of death in Lebanon and the second in Qatar after road injuries [11]. Both countries included prevention strategies against chronic diseases generally and CVD especially in their national vision and had set future plans to manage and control the spread of cardiovascular diseases [12–14]. Moreover, the medicine prices were perceived to be high in these two countries compared to respective neighboring countries [15–17]. Considering this evidence, Abdel Rida et al. conducted a narrative review and document analysis of the pharmaceutical pricing policies in Qatar and Lebanon [18]. Both Qatar and Lebanon have implemented similar pharmaceutical pricing policies. External reference pricing (ERP) is one of the pricing policies adopted by both countries, albeit with different baskets of reference countries. The basket of countries to which the prices in each country are benchmarked vary [19, 20]. In addition to ERP, mark-up regulations are applied with different schemes along the pharmaceutical supply chain. While a decree detailing all the different regressive mark-up^1^ schemes is available to the public in Lebanon [21], such detailed scheme is not available in Qatar. The standardized mark-up is linear and capped at 44% on all medicine in the private sector. In addition, Qatar may consider the economic evaluation of a medicine to set the price if such evaluation or Health Technology Assessment (HTA) is available at the time of registration [20]. The prices of generic medicines are set at fixed lower percentage in comparison to the price of the originator brand in both countries as per Appendix. Generic brands are classified according to the chronological order in which they get registered and enter the market in both Qatar and Lebanon [19, 20]. The price of the first registered generics is 30 and 35% lower than their comparative OB available in the market in Lebanon and Qatar respectively. Additionally, pricing of generics in Lebanon considers the country of origin in a pricing strategy and the price variation of different generic brands within the same category similar to internal reference pricing used internationally [19, 22]. Lebanon also adopts yet a different markup scheme for locally manufactured generics [19].

Qatar and Lebanon differ in terms of economic indicators due to the differences in income levels. While Qatar is a high-income country, Lebanon is an upper-middle income country. The pharmaceutical sector is a major subset of the health sector especially with spending on pharmaceuticals constituting a high percentage of the total health expenditure [23–25]. In Lebanon, almost half of the health expenditure is attributed to purchase of pharmaceuticals [26] while it only constitutes 10.90% of the total health expenditure in Qatar where most of the expenditure on health was in the public sector as per Table 1 below. In the last decade, the public health sector in Qatar has been expanding to provide high-standard primary health care services across the state. The health services are provided for free for Qatari citizens while residents or non-Qatari pay 20% of the total healthcare bill. The primary health care centers in Lebanon cover most of the Lebanese territory, however, due to budget constraints, services are limited in terms of population coverage, clinical services and pharmaceutical products dispensing. If available, medicines are dispensed free of charge for beneficiaries in the public primary health centers in Lebanon. Out of an approximate population of 4.5 million in 2011, only 162,016 patients had access to chronic non-communicable diseases medicines from the public sector in Lebanon [17].

**Table 1:** Qatar and Lebanon economic indicators and demographics

	Qatar	Lebanon
GDP (bn)	$164.64	$47.08
Population (mn)	2.58^a^	6.24^b^
*Citizens* (mn)	*0.313*^c^	*4.751*^b^
GDP Per Capita (35)	$73,653	$8,047
Health Expenditure (bn)	$4.82	$3.34
*Public Health Expenditure* (bn)	*$4.10*	*$1.59*
*Private Health Expenditure* (bn)	*$0.72*	*$1.75*
Pharmaceutical Sales (bn)	$0.52	$1.64
Pharmaceutical Sales, % of Health Expenditure	10.90	49.00
Per Capita Spending on Pharmaceuticals	$234	$280
Spending on Originator Pharmaceuticals (mn)	$360.0	$800.0
Spending on Generic Pharmaceuticals (mn)	$110.0	$490.0

*Source:*

World Bank [27]

Business Monitor International (BMI) [26]

Business Monitor International [28]

^a^Ministry of Development Planning and Statistics (mdps) [29]

^b^Central Intelligence Agency (CIA) [30]

^c^Snoj [31]

The management and control of the spread of CVD starts with a prevention plan, however, treatment is equally essential. While acknowledging the efforts of both countries to implement pricing guideline (e.g., benchmarking and mark-up), it is imperative to analyze the status of essential medicines (i.e. the access to essential medicines in terms of price, availability, affordability, and accessibility) aimed at treating CVD which are highly prevalent. Assessing access to these medicines is needed in order to achieve optimal adherence to secondary prevention measures in treating early stages of CVD, to minimize complications, hospitalizations, and mortality, and to control the total cost of diseases management [23]. Level of access to medication is determined through the evaluation of prices, availability and affordability of these medicines [32]. Thus far no research has been conducted in Qatar to investigate whether all essential CVD medicines are available or affordable in the private or public sector especially after the phased price cut undertaken by the gulf countries [33]. In this phased price cut, medicines were classified into five main categories and the prices within each category were reviewed and reduced [33]. Similarly, the latest survey conducted in Lebanon by the WHO/HAI included only three CVD medicines [17] and was published in 2013 prior to the latest review of pharmaceutical pricing guidelines at the end of 2014 [21]. The main objective of the WHO/HAI survey is to collect reliable data related to prices, availability, and affordability of a specific basket of medicines and price components added throughout the supply chain. Given the profile of CVD and the pharmaceutical pricing containment strategies undertaken in Qatar and Lebanon as presented above [19–21], this study was developed to collect data from different sectors and medicine selling outlets (i.e. community pharmacies or outpatient pharmacies), in order to permit a better understanding of the current pharmaceutical situation in terms of cardiovascular disease medicines price, availability, and affordability in both countries.

## Methods

### Study methodology overview

Ideally, WHO/HAI methodology is used for a national survey, or across a state or province in case of large countries. Data about the medicines’ price, availability and affordability are collected from up to 4 different sectors. It is recommended to cover at least the capital plus 5 other areas within one-day drive from the capital. In each area, it is recommended to collect data from 5 outlets per sector. In case of an outlet with an availability of medicines less than 50% of the list surveyed, another outlet within the same area should be visited. Moreover, validation visits for 20% of the outlets is recommended. Lebanon and Qatar have comparable areas, 10,452km^2^ and 11,610km^2,^ respectively [34]. However, the populations vary significantly. Lebanon has a population of more than 5 million, almost double the population of Qatar with a much higher population density [34]. According to WHO/HAI [9], the selection of the covered areas should be based on geographical districts around the country with a minimum population density. The real retail price paid by the patient is collected in each sector in local currency. Surveyed outlets should have an outpatient pharmacy dispensing medicines directly to the patient. To calculate the affordability, the lowest-paid unskilled government worker (LPGW) salary is identified. Affordability is then estimated based the cost of a full treatment regimen for either acute or chronic disease in terms of the number of the days’ wages forgone by the LPGW to purchase the treatment. The outlet sampling is conducted in a randomized and systematic manner to ensure enough coverage. The selection of outlets is related to the demographics of a given area surveyed. The purpose is to randomly choose a region and consequently outlets that comply with the criteria set by WHO/HAI e.g.: minimum population and distance from the capital. The list of surveyed medicines can include up to 50 medicines while the affordability can be calculated for up to 22. For each medicine, data about the originator brand (OB) and lowest-priced generic (LPG) available at the outlet are collected. The second edition of *Measuring medicine prices*, *availability*, *affordability*, *and price components* (2008) of WHO/HAI methodology was adopted with some variations in terms of number of areas covered in Qatar; facility number per sector and area; back-up and validation visits. This was due to the inherent geographical and demographic characteristics of the countries surveyed as mentioned above especially in Qatar and to the limited resources available to the researchers.

### Study design

The study was a cross-sectional observational and comparative survey, carried out in two different countries: Qatar and Lebanon. The main areas surveyed were the capital cities in both countries (Doha and Beirut) in addition to other administrative areas within one-hour drive from each capital. In Lebanon, the population is dispersed across the country. However, Qatar has a centralized population density around fewer main cities or municipalities. As such, the availability and types of healthcare services and facilities in both countries are reflective of the demographic profiles. The 1 + 5 target was achievable in Lebanon due to its multiple population centers [9]. However, the centralized population in Qatar limited our surveyed areas to 1 + 4.

### Population sampling and data collection

Data were collected from public health care centers (Public Sector), private pharmacies (Private Sector) in both countries and private hospitals or clinics (Other Sector) exclusively in Qatar between November 2016 and April 2017. No private hospitals/clinics were covered in Lebanon since none had an outpatient pharmaceutical dispensing outlet. In each area surveyed, a public outpatient health care center was identified, and thereafter, a proximal private sector facility was selected within the same region. The medicine outlets surveyed were contacted and informed of the date, time, and approximate duration of the visit. One facility per sector was covered.

In case of an outlet where less than 50% of the surveyed medicines were available, a back-up outlet from the same sector was visited where possible. Finding a back-up private community pharmacy in both countries was possible. However, this was not an option in the public sector in Qatar given that only one primary health care center is available per specific area. No validation visits were conducted. For each medicine, data about the originator brand (OB) and the lowest-priced equivalent generic (LPG) available in the outlet at the time of the visit were collected. Each outlet was visited once.

### Medicine selection

**Fig. 1:**
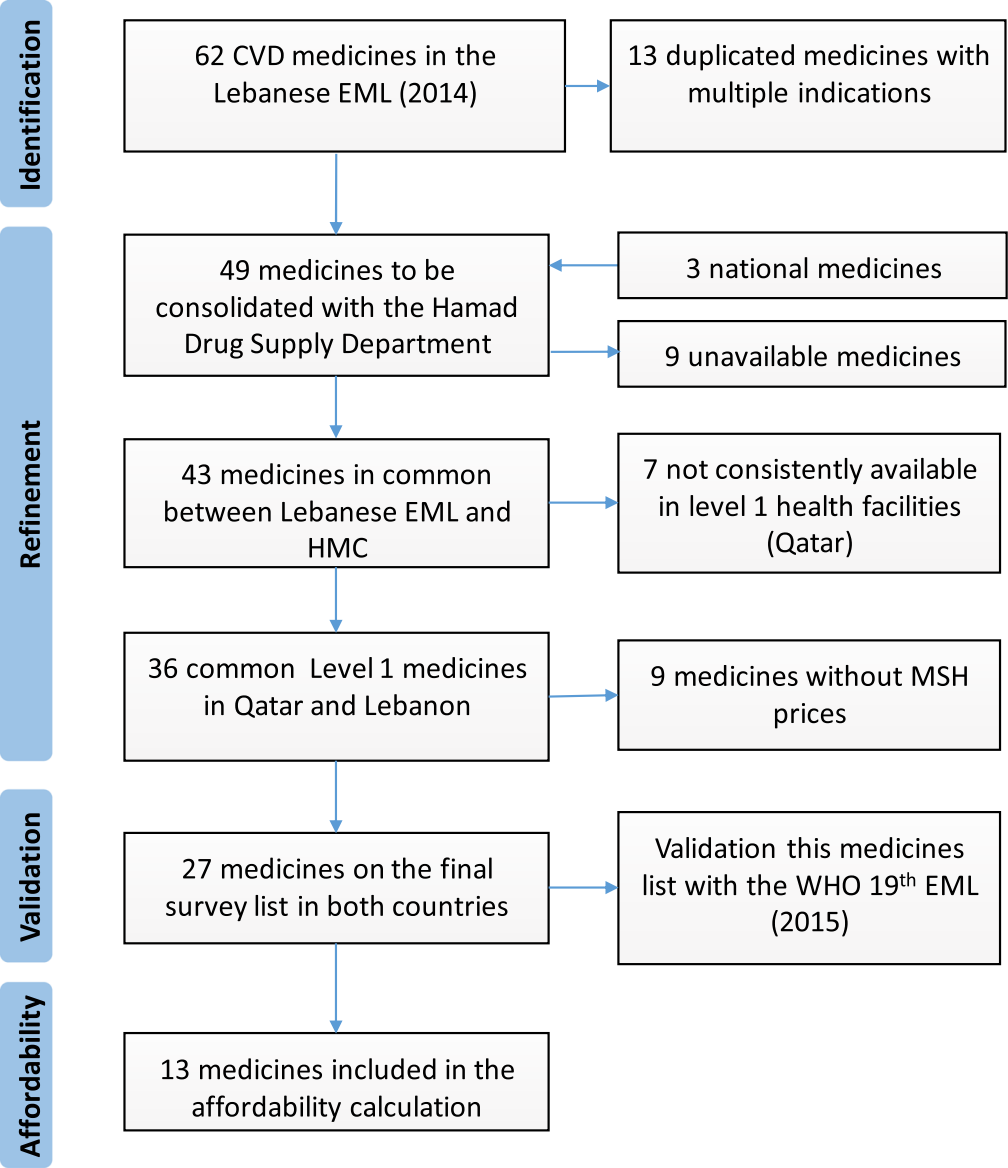
Refinement process of the surveyed list of medicines

To compile a common list of essential CVD medicines, the 2014 Lebanese essential medicine list (EML) available at the time of the study was adopted [35]. This list was in line with the 18th edition of WHO list [35]. To compile a common list, the CVD medicines were first compared to the medicines available in Qatar. As a result, the list of Lebanese CVD essential medicines was refined by excluding the medicines that were not available in Qatar, were not available in level 1 facility, or did not have an international reference price (IRP) as per Fig. 1. The 27 common essential CVD medicines achieved were compared to the latest WHO 19th EML (Table 2). Given that only 22 medicines can be considered for affordability calculation as per the WHO/HAI methodology, only the first line treatment medicines for the main cardiovascular diseases were included in our survey. The decision was made after reviewing the latest cardiovascular disease clinical guidelines [36] and consolidated by a meeting with the clinical pharmacy department in Hamad Medical Corporation (HMC) in Qatar. The final medicine considered for the affordability calculation covered those used for one or more of the following diseases: angina, arrhythmia, hypertension, hyperlipidemia, heart failure, and finally antithrombotic medicines [35]

**Table 2:** List of medicines surveyed

Medicine name	Dosage strength	Dosage form	Affordability
Acetylsalicylic acid	100 mg	cap/tab	*✓*
Amiodarone	200 mg	cap/tab	*✓*
Amlodipine	5 mg	cap/tab	*✓*
Atenolol	50 mg	cap/tab	
Atenolol (2)	100 mg	cap/tab	
Atorvastatin	10 mg	cap/tab	
Atorvastatin (2)	20 mg	cap/tab	*✓*
Atorvastatin (3)	40 mg	cap/tab	
Bisoprolol	5 mg	cap/tab	*✓*
Captopril	25 mg	cap/tab	
Captopril (2)	50 mg	cap/tab	
Clopidogrel	75 mg	cap/tab	*✓*
Digoxin	0.25 mg	cap/tab	*✓*
Diltiazem	60 mg	cap/tab	
Enalapril	5 mg	cap/tab	*✓*
Furosemide	40 mg	cap/tab	*✓*
Gemfibrozil	600 mg	cap/tab	
Hydrochlorothiazide	25 mg	cap/tab	*✓*
Isosorbide dinitrate	5 mg	cap/tab	
Losartan	50 mg	cap/tab	
Methyldopa	250 mg	cap/tab	
Propranolol	10 mg	cap/tab	
Propranolol (2)	40 mg	cap/tab	
Simvastatin	10 mg	cap/tab	
Simvastatin (2)	20 mg	cap/tab	*✓*
Spironolactone	25 mg	cap/tab	*✓*
Verapamil	80 mg	cap/tab	*✓*

### Outcomes measures

Unit prices of either tablets or capsules in local currency were recorded. To calculate the median price ratio (MPR), the unit price were converted to US dollar as per *Oanda* [37] then divided per the IRP collected from International Price Guide (2014) published by Management Sciences for Health (MSH) [38] as per the equation below.

$$ MPR\  of\ a\  specific\ medicine=\frac{median\ local\ unit\ price}{international\ reference\ unit\ price} $$

Most of the data collection took place between August–November 2016. As the public primary healthcare centers survey in Qatar was surveyed in 2017, hence the median price ratios for this sector were adjusted based on the corresponding year’s consumer price index (CPI) [39]. The median price ratio was calculated if a medicine was found in at least three outlets per sector. An MPR of maximum 4 was considered as a threshold in this study given the costs at different stages along the supply chain. For international comparison of MPR, prices collected in both countries were adjusted to purchasing power parity (PPP) as directed in the WHO/HAI manual [9], conversion factors were collected from World Bank [27]. Availability is the percentage of facility where a medicine is stocked out of the total number of facilities per sector. Finally, affordability is expressed in the number of days’ wage forgone to purchase the complete treatment of a specific medicine for a month [9]. The daily wages for the lowest paid unskilled government workers (LPGW) were $20 according to the laws in Qatar and Lebanon [40, 41]. Only medicines that were either first choice for each cardiovascular disease or those that are of national clinical priority were considered for the affordability calculation. The treatment regimen was based on the international standard regimen as per WHO [9] and the new national clinical guidelines launched by the Ministry of Public Health in Qatar [36].

### Data analysis

The Excel workbook available on the HAI website (www.haiweb.org/medicineprices) was utilized for data entry and summary. Daily management and review of collected data were conducted to minimize missing data and errors.

### Ethics

No institutional review board (IRB) approval was required for the study as confirmed by Qatar University. Prior to surveying the “public sector”, ethics approval was received from the Primary Health Care Corporation (PHCC) in Qatar and a letter directed to the General Manager of the Ministry of Public Health in Lebanon seeking approval was accepted and signed. As for the private sector, a letter of support from Qatar University was issued and shared with the pharmacy managers prior to the survey in Qatar. In Lebanon, an endorsement phone call from the local study collaborator was made before the visits.

## Results

Due to the inherent characteristics of the two countries surveyed and limited resources, only two different sectors were surveyed in Lebanon and three in Qatar.

Similarly, the number of facilities visited per sector and per area was limited to one or two upon availability. A total of 30 outlets were visited, 15 per each country.

### Prices

#### Price variation in local currency

More price variation incidences were observed in Lebanon than in Qatar of the same medicine type (either OB or LPG) between different outlets within and across sectors. For instance, the MPR of Atorvastatin 20 mg (LPG) in Qatar demonstrated a slight variation between 27.02 and 30.47, while the MPR values of the LPG brands in Lebanon varied between 9.96 the lowest and 18.51 the highest. For this same medicine, the median MPR in Qatar was approximately twice the figure in Lebanon (29.06 vs. 15.34). The price variation of LPG was higher than those of OB in line with the multitude of generic brands for the same medicine found in the outlets especially in Lebanon. Table 3 demonstrates the few instances of these variations.

**Table 3:** MPR percentiles variation in the private sectors for both medicine types in Qatar and Lebanon

Country/sector	Medicine and product type	Median MPR	25th percentile	75th percentile
Qatar/ Other Sector	Acetylsalicylic acid- OB	12.07	11.91	12.07
	Atorvastatin 20 mg- OB	24.11	24.11	24.11
	Atorvastatin 20 mg- LPG	29.06	27.02	30.47
Lebanon/ Private Sector	Atorvastatin 20 mg- LPG	15.34	9.96	18.51
	Hydrochlorothiazide 25 mg- OB	14.25	13.43	15.07
	Captopril 50 mg- OB	3.83	3.66	3.83

#### Median price ratio (MPR)

Table 4 illustrates the median MPR for both medicine types across sectors in Qatar and Lebanon. Medicines are free-of-charge in the public sector in Lebanon and only for the Qataris in Qatar. Only the “Public Sector Patient Prices” in Qatar showed acceptable MPR with a median CPI-adjusted MPR of 1.44 (0.45–2.33) and 0.01 (0.01–0.01) for OBs and LPG, respectively. The LPG CPI-adjusted MPR is not representative of all generics in PHCC since sufficient data was only available to calculate the median MPR of captopril 50 mg. The median MPR in the private sector and other sector patient prices were comparable for each medicine type. However, median MPRs for these two sectors were significantly higher than those of a comparative basket purchased from PHCC (Table 5). Purchasing a comparable basket of medicines from community pharmacies or from outpatient pharmacies, patients would have to pay 13.6 and 14.2 times the price paid in PHCC. The median MPR of medicines dispensed in the community pharmacies in Lebanon is 12.37 (*IQR* = 3.30–32.57) for OB and 5.82 (*IQR =* 1.85–13.56) for LPG, whereas in Qatar it is quite comparable with 21.60 (IQR = 6.42–40.43) vs. 26.50 (IQR = 22.70–29.26) for OBs and LPGs respectively (Table 4). It is important to mention that an MPR of more than 5 for the private sector and more than two for public sector is considered as a cut-off values for fairly high prices.

**Table 4:** Median CPI-adjusted MPR of originator and generic brands in all sectors surveyed in Qatar and Lebanon

	Qatar						Lebanon	
	*Public (n = 5)*		*Private (n = 6)*		*Other (n = 4)*		*Private (n = 6)*	
	OB	LPG	OB	LPG	OB	LPG	OB	LPG
Median MPR (IQR^a^)	1.44 (0.45–2.33)	0.01 (0.01–0.01)	21.60 (6.42–40.43)	26.50 (22.70–29.26)	26.71 (14.44–46.04)	29.52 (25.11–33.81)	12.37 (3.30–32.57)	5.82 (1.85–13.56)
Min MPR	0.09	0.01	0.72	0.40	0.72	13.28	0.52	0.32
Max MPR	9.68	0.01	67.73	44.10	67.73	44.10	59.73	41.13
Medicines included	23	1	19	9	14	4	20	17

Note: *Min* minimum, *Max* maximum

^a^Interquartile range

**Table 5:** Comparison of the median MPR between a common basket of matched pair of medicines across sectors in Qatar

	Public	Private	Public	Other	Private	Other
Number of matched medicines	17		14		14 (OB) 4 (LPG)	
MPR - OB	1.77	24.11	1.89	26.71	26.71	26.71
MPR - LPG					27.92	29.72
% Difference	1264.90%		1316.90%		0.00% OB/ 6.40% LPG	

#### International comparison

After adjusting for purchasing power parity, the median MPR of medicines (both types) revealed higher price ratio to international reference prices in Qatar than in Lebanon (Table 6). Table 7 demonstrates few examples of purchasing power parity (PPP) and consumer price index (CPI) adjusted-MPR.

**Table 6:** PPP and CPI-adjusted median MPR in all sectors surveyed in Qatar and Lebanon

	Qatar						Lebanon	
	*Public (n = 5)*		*Private (n = 6)*		*Other (n = 4)*		*Private (n = 6)*	
	OB	LPG	OB	LPG	OB	LPG	OB	LPG
Median MPR (IQR)	2.75 (0.86–4.46)	0.03 (0.03–0.03)	41.36 (12.30–77.43)	50. 78 (43.46–56.03)	51.15 (27.66–88.17)	56.92 (48.09–64.75)	21.39 (5.71–56.29)	10.06 (3.20–23.43)
Min MPR	0.18	0.03	1.38	0.76	1.38	25.43	0.90	0.55
Max MPR	18.53	0.03	129.71	84.45	129.71	84.45	103.23	71.09
Medicines included	23	1	19	9	14	4	20	17

**Table 7:** PPP and CPI-adjusted MPR of individual medicines in community pharmacy

Medicine	Qatar		Lebanon	
	OB	LPG	OB	LPG
Clopidogrel 75 mg	70.34	43.46	37.61	13.72
Atorvastatin 20 mg	89.72	48.76	60.75	26.52
Amlodipine 5 mg	129.71	84.45	54.55	44.37
Acetylsalicylic acid 100 mg	23.12		18.15	12.94

#### Brand premium

Across all four sectors, the MPR of lowest priced generics were lower than those of their respective originator brands (Table 8). With the near absence of LPG in the public sector in Qatar, the prices of matched LPGs were almost 39 and 34% lower than their corresponding originators in the two private sectors surveyed. In Lebanon, for the matched pairs of medicines found (13 medicines), the price of LPGs was 65% less than their respective OBs (Table 8).

**Table 8:** MPR of comparable basket of matched OB and LPG pair medicines found per sector

	Qatar						Lebanon		
	*Private*			*Other*			*Private*		
	OB	LPG	Brand prem.	OB	LPG	Brand prem.	OB	LPG	Brand prem.
	9	9		4	4		13	13	
Median MPR	43.61	26.52	1.64	45.23	29.72	1.52	21.76	7.64	2.85
25%ile MPR	36.73	22.70	1.62	38.73	25.11	1.54	4.81	2.15	2.24
75%ile MPR	48.28	29.26	1.65	52.07	33.81	1.54	35.15	15.34	2.29
Min MPR	0.72	0.40	1.80	24.11	13.28	1.82	0.68	0.32	2.13
Max MPR	67.73	44.10	1.54	67.73	44.10	1.54	59.73	41.13	1.45

Note: *Min* minimum, *Max* maximum, *Prem.* premium

As reported per the Qatari and Lebanese pharmaceutical pricing guidelines [19, 20], generic medicines should be priced at least 35 and 30% less than the originator brands in Qatar and Lebanon, respectively. By referring to the brand premium observed in our survey (Table 8), prices of LPG are in line with the regulations in both countries.

### Availability

A high variability in the mean availability of originator brands and lowest priced generics were detected especially in the public primary health centers in both countries (Table 9). The mean availability of generics in Qatar is low with its highest mean (SD) of 29.60% (37.10%) observed in the community pharmacies. In the same context, Qatar relies mainly on OB in its PHCC centers where mean availability (SD) of OB was 82.20% (27.90%) in the 5 outlets visited with a minimal supply of LPG 5.90% (15.50%). PHC in Lebanon are only dispensing generic essential medicines, nevertheless, the availability was critically low with only 46.90% (36.10%). The mean availability of OB and LPG medicines in the community pharmacies in Lebanon was higher than the two private sectors in Qatar. In Lebanon, patients were more likely to face low availability of essential CVD medicines in the public sector in comparison to the private pharmacies.

**Table 9:** Mean availability (in percent) of cardiovascular diseases medicines in sectors surveyed in Qatar and Lebanon

	Qatar						Lebanon			
	Public (*n* = 5)		Private (*n* = 6)		Other (*n* = 4)		Public (*n* = 9)		Private (*n* = 6)	
	OB	LPG	OB	LPG	OB	LPG	OB	LPG	OB	LPG
Mean Avail.	82.20	5.90	64.80	29.60	54.60	25.00	0.00	46.90	69.80	58.60
SD	27.90	15.50	37.90	37.10	40.50	34.70	0.00	36.10	32.70	39.90

Note: *Avail.* availability, *SD* standard deviation

### Affordability

Affordability was calculated for those medicines available in three or more outlets per sector. Medicines were affordable in the public primary healthcare centers in Qatar (Table 10). While nine out of thirteen medicines were at 1 days’ wage or less in all private sector outlets surveyed in both countries, enalapril (antihypertensive medicine), simvastatin and atorvastatin (lipid-lowering agents), and clopidogrel (antithrombotic) monthly regimen exceeded the threshold. The originator brand of clopidogrel was the most burdening medicine in both countries as LPGWs have to forgo 4.2 and 2.5 days’ wages in Qatar and Lebanon, respectively, in order to purchase their monthly dosage from the private sectors.

**Table 10:** Affordability in number of days’ wages needed to purchase standard treatment

	Days’ wages needed to purchase a 30 days’ treatment			
	Qatar			Lebanon
	PHCC	Community pharmacies	Private clinics/hospitals	Community pharmacies
Bisoprolol 5 mg × 60	0.1 OB	0.9 OB	0.9 OB	1 OB/ 0.4 LPG
Digoxin 0.25 mg × 30	0 OB			0 OB
Verapamil 80 mg × 90	0.2 OB			1 OB
Amiodarone 200 mg × 30	0.1 OB			0.3 OB / 0.2 LPG
Amlodipine 5 mg × 30	0.1 OB	0.9 OB / 0.6 LPG	0.9 OB / 0.6 LPG	0.4 OB / 0.4 LPG
Enalapril 5 mg × 120	0.1 OB	3 OB / 1.6 LPG	3 OB	
Hydrochlorothiazide 25 mg × 30	0 OB	0.3 OB	0.3 OB	0.2 OB
Furosemide 40 mg × 30	0 OB	0.4 OB	0.4 OB	0.4 OB
Spironolactone 25 mg × 30		0.4 OB		0.2 OB
Acetylsalicylic acid 100 mg × 30	0 OB	0.1 OB	0.1 OB	0.1 OB / 0.1 LPG
Clopidogrel 75 mg × 30	0.4 OB	4.2 OB / 2.6 LPG	4.2 OB	2.5 OB / 0.9 LPG
Simvastatin 20 mg × 30	0.1 OB	1.4 OB / 1.1 LPG		1.4 OB / 0.2 LPG
Atorvastatin 20 mg × 30	0.3 OB	3.1 OB / 1.7 LPG	3.1 OB / 1.9 LPG	2.3 OB / 1 LPG

For originator brand medicines that impose high burden on LPGW, cheap affordable generics existed. For instance, by substituting originator branded clopidogrel by its generic equivalent, LPGW could have saved 1.6 days’ wages in Qatar and Lebanon respectively.

## Discussion

This study was planned to assess the price, availability and affordability of essential cardiovascular medicines within and across Qatar and Lebanon. This study found out that the medicine price was more uniform across the same sector in Qatar than in Lebanon. In the Qatari public sector, medicines were priced lower than the international reference prices. However, the prices of medicines in the private sector were higher than the international reference prices in both countries. The MPR of OBs and LPGs in the private sectors in Qatar (private sector, and other sector surveyed) were up to two and five times higher than those in Lebanon, respectively. In terms of availability, only the public sector in Qatar met the WHO target for OB CVD medicines. Medicines in the public sector were dispensed free-of-charge for beneficiaries of the National Chronic Drugs Program in Lebanon and for Qatari nationals in Qatar and were all affordable for non-Qataris in the primary health care centers (public sector). In the private sectors surveyed, there were medicines considered unaffordable. In cases where the OB was not affordable, a less expensive generic substitute was available. The higher occurrence of the price variations of the originator brands observed in Lebanon could be due to the fact that the medicine price may change every 2 weeks according to the bi-monthly index released by the MoPH to all warehouses and pharmaceutical outlets. These indices adjust prices based on foreign currency exchange rates fluctuations [17]. Small variations are commonly encountered [42] even under strict pharmaceutical pricing and monitoring regulation as in Saudi Arabia [43], Iran [44] and Lebanon in 2013 [17]. As for LPG’ price variations, this could be attributed to the fact that several generic brands of the same active ingredient exist in the market as observed in Saudi Arabia in 2015 [43], Lebanon 2013 [17] and Iran 2014 [44].

The median MPR of OB prices paid by the non-Qatari residents in the public sector were the lowest when compared to those in countries of any income level including Sri Lanka [45], Iran [44], and 11 other Asia Pacific countries [46]. The significant difference in the prices paid by patients between the public and private sectors in Qatar demonstrated the Qatari vision for a subsidized public healthcare system that is of international standard and affordable to all. The high ratios to IRP in community pharmacies in Qatar (21.6 for OB and 26.52 times the IRP for LPG) were also significantly higher when compared to Saudi Arabia (6.66, 8.88 times the IRP) [43] and Iran (3.62, 1.21 times the IRP) [44].

Given that Saudi Arabia and Qatar are utilizing the same pharmaceutical pricing guidelines and bulk procurement for the private sector, the difference in MPR could be related to the higher and linear mark-up schemes applied in Qatar compared to a regressive mark-up in Saudi Arabia. In Lebanon, medicine prices were reduced by the end of 2015, the median MPR of OB increased in 2016 by 2.5% (12.36 vs. 12.06 times the IRP). This can be due to the fact that the 2013 WHO/HAI survey included a different basket of chronic and acute diseases essential medicines whose prices may have affected the overall median MPR [17]. As for generics, their prices decreased by 18.2% (5.48 vs. 6.7 times the IRP and close to threshold) after the pricing mechanism of generic medicines was reevaluated, however it is still significantly higher than Iran (1.21 times the IRP) most likely due to more developed domestic generic manufacturing in Iran [47, 48].

Accounting for the difference in economic strength [27, 49] did not reduce the gap of difference in price observed between Qatar and Lebanon which is in line with other studies that showed the absence of any link between the level of income and the prices of medicines in a country [50–52] except for Schweitzer and Comanor study [53]. The price difference between low-middle-income countries (LMIC) or developing countries on one hand and industrialized countries on the other hand could be due to differential pricing [54, 55].

Given that the adjustments to prices applied in this study did not provide possible explanation for the gap in prices between Qatar and Lebanon, further investigation of the landed price^2^ and the pricing mechanisms is needed in the future to provide answers [9].

With the international WHO target to achieve more than 80% availability of affordable essential generic medicines in at least the public sector by 2030 [1], a slight improvement of the availability of generic essential CVD medicines was observed in the Lebanese public sector in comparison to the year 2013 (46.9% in 2016 vs. 42% in 2013). However, this availability is lower compared to the upper-middle income countries (UMIC) average as per Ewen et al. study [1]. This discrepancy may be related to the difference in the medicine basket surveyed.

The absence of OB of essential CVD medicines in the public sector in Lebanon is in line with what Ewen et *al*. (2017) demonstrated in their secondary study of WHO/HAI between 2008 and 2015 for UMICs surveyed in which Lebanon was included alongside Iran, Colombia, Ecuador, Mauritius, Mexico, and Brazil [1].

The subsidy of the public sector in Qatar was also reflected in the predominant availability of originator brands of the surveyed medicines. This availability exceeded the WHO target (82.2%) [1] and is significantly higher than OB NCD medicines in the same sector in Saudi Arabia (82.2% vs. 22.3%) [43] and the generic medicines availability in the public sector in Iran (75.2%) [44], where pharmaceutical pricing policies were enacted to control and challenge the OBs entry into the market while generics manufacturing is subsidized and their prices are controlled and competitive [47, 48]. The low availability of generic medicine in all sectors in Qatar may be related to consumer preferences for OBs [56, 57]. The unavailability of any type of digoxin, diltiazem, amiodarone or isosorbide dinitrate in the private sector may be related to their usage restriction in community pharmacies and to the structure of the healthcare system in Qatar where private cardiology medical services in Qatar are generally limited and mostly available in major hospitals in the capital city.

Our results showed that medicines in the public sector in Qatar were affordable to LPGW. Compared to the private sector in Saudi Arabia, purchasing of medicines by non-nationals in the private sector in Qatar is more affordable when comparing all types of amlodipine and clopidogrel [43]. In Lebanon, despite the fact that the OB medicines exceeded the one-day’ wage threshold in 2016, the few common essential CVD medicines surveyed in Lebanon (clopidogrel, amlodipine, amiodarone, hydrochlorothiazide, simvastatin and atorvastatin) became more affordable in 2016 compared to 2013 [17] and were more affordable than in Saudi Arabia (2015) for any type [43], and more affordable than the OBs in Iran (2014) [44]. Once more, the pharmaceutical pricing policy of OBs in Iran may have resulted in more expensive OB medicines in comparison to Lebanon.

Nevertheless, the official LPGW salaries adopted for calculation in both Qatar and Lebanon omit a substantial portion of the local population. In Qatar, out of a population of circa 2.58 million, around one million are blue-collar laborers employed in the private sector that are paid almost half of the LPGW salary adopted in our study. In order to calculate affordability for these workers, we would need to almost double the number of days’ wages required to purchase a treatment course. Similarly, according to the 2016 UNDP report on Lebanon, 27% of the population is living under the poverty line and earning less than $4 per day [58]. Moreover, as of 2011, there were only 162,016 out of registered beneficiaries under the National Chronic Drug Program in Lebanon who are eligible to obtain free medicines in primary healthcare centers [17]. Overall, roughly half of the Lebanese population has some form of health coverage (public and private) [17], while in Qatar all residents are eligible for subsidized healthcare services and medication through the Primary Health Center Corporation (PHCC) and Hamad Medical Center (HMC). Considering the low availability of medicines in the public sector in Lebanon, most of the patients with chronic diseases are purchasing their medicines from the private sector and thus paying out-of-pocket for CVD medicines. It is worth mentioning that patients with pre-existing conditions or chronic diseases can sometimes find it difficult to obtain private insurance as insurers can significantly increase premiums and deductibles or refuse coverage to such patients. It is important to note that most CVD cases require multidrug regimens [2], hence the affordability of a treatment plan would depend on the sum of prices of all drugs prescribed to a patient. The study findings provide several possible policy actions that could be adopted to improve sustainable access to affordable medicines.

Such studies provide valuable advocacy messages for policymakers, pharmaceutical industries, regulators, prescribers and patients if well shared and delivered in a timely manner. Generic uptake in Qatar is still at its infancy, and more effort could be made to promote the acceptance of generic brands among prescribers, pharmacists and patients. Pharmacist should be legally permitted to substitute generic brands for OB. As such, an amendment to the pharmacy practice law enacted in 1980 must be considered. Moreover, facilitating the regulatory process involved in the registration of prequalified quality generic and encouraging the local manufacturing of such medicines are highly recommended. This would result in the optimization of the drug budget in the Qatari public sector by procuring more good quality generic medicines and by providing a cheaper alternative for patients in the private sector.

The price of medicines in the private sector in Qatar must be reconsidered for further price reduction and the linear mark-up added to the supply chain should be replaced by a regressive scheme. Several policy actions could be implemented in Lebanon as well. First, MoPH should prioritize the pharmaceutical budget to ensure that all essential medicines on the national EML are available in the public sector. This can increase the availability of medicines in the public sector and decrease the out-of-pocket spending on pharmaceuticals in the private sector. The coverage of NCD patients under the National Chronic Drugs Program must also be expanded to include the growing segment of the population below the poverty line. The quality of the available generic medicines especially in the private sector in Lebanon should be rigorously assured, and more emphasis should be put into the regulatory process prior to registration to avoid substandard and counterfeit medicines.

In light of the recent regional blockade on Qatar, it would be relevant to reconduct the survey to measure the efficiency of measures undertaken by local authorities to overcome the situation. More frequent surveys of most common communicable and non-communicable diseases medicines and their price components are needed. The data on medicine price collected in our survey could be used by other methods that measure affordability such as impoverishing and catastrophic effects.

The design of the survey is cross-sectional while in countries where pharmaceutical policies and pricing are amended over time, changes may be better detected by longitudinal studies. Strategic and logistic limitation may have affected the findings. Thus, two limitations arose; a sample size that might have affected the external validity of the study findings and the fact that no quality analysis of medicines was conducted. Furthermore, the affordability may have been overestimated by neglecting the multidrug regimens fact and other healthcare expenses. Finally, including only essential medicines in the surveyed list may have been restrictive especially for the private sector.

To the best of our knowledge, this study is the first in Qatar and the fifth in a high-income country (HIC). Second, the medicines surveyed are of global and national priority and are proven to be safe and cost-effective. Our findings could also be used as indicators for the assessment of millennium development targets and other NCD indicators. Key policy decisions should be implemented to improve access to medicines in both countries.

## Conclusion

By using a variant of the WHO/HAI methodology, we quantitatively evaluated the medicine prices in comparison to international reference prices, as well as their availability and affordability in both Qatar and Lebanon. Pharmaceutical pricing policies were also summarized for comparative purposes. In summary, data revealed that medicine prices were more uniform across the same sector in Qatar than in Lebanon. In general, the price of originator brands and lowest priced generics in Qatar were higher than those in Lebanon. Across all four sectors, the MPR of LPGs were lower than those of their respective OBs. Generally, the studied medicines in the public sector in both countries were either free-of-charge or affordable. In the private sectors surveyed, some medicines were consistently unaffordable. Even in cases where the OB was not affordable, a less expensive generic substitute was available. In terms of availability of medicines, only the public sector in Qatar met the WHO recommendation. In addition, medicines were more available in the private sector in Lebanon than in Qatar. The originator brand availability in the public sector in Qatar exceeded the WHO recommendation. Except for the public sector in Qatar, both countries fall short of the Sustainable Development Goals, and more efforts should be undertaken to achieve these goals.

## Data Availability

The datasets generated and/or analysed during the current study are available in the QSpace repository, https://qspace.qu.edu.qa/handle/10576/11343. It is also available from the corresponding author on reasonable request.

## Appendix

**Table 11:** Generic brands pricing strategies in comparison to OB in Qatar

Qatar	
First generic	-35% of the OB price
Second until fourth chronologically registered generics	-10% from the last registered generic
Fifth generic onward	Same price as the fourth registered generic in the market
Locally manufactured under licensed or locally packaged generics	Same pricing mechanism as imported generics

**Table 12:** Generic brands pricing strategies in comparison to OB in Lebanon

Lebanon			
Generics with available OB in the market	-30% of the OB price		
Generics with no available OB in the market	Category 1	Country of origin: US, UK, Canada, France, Austria, Belgium, Sweden, Germany, Switzerland, Ireland, Norway, Denmark, Italy, Spain, Netherlands, Finland, Japan, and Australia	Price for the first three generics in each category is set based on Price Certificate presented at registration. Within 3 months of a new generic being registered, the price is revised to be the average price of all generics within the category. Price is also revised in case of price variation of any of the generics in the category.
	Category 2	All other countries	
Locally packaged generics (under license)	Price depends on the ex-factory price in the country of origin	In both cases, price should be 30% less than OB	
Locally manufactured generics	Price depends on local ex-factory price		

## Abbreviations

CPI: Consumer price index
CVD: Cardiovascular diseases
EML: Essential medicine list
EMRO: Eastern Mediterranean Regional Office of the WHO
HIC: High-income country
HMC: Hamad Medical Center
HMC: Hamad Medical Corporation
IQR: Interquartile range
IRB: Institutional review board
IRP: International reference price
LMIC: Low-middle-income countries
LPG: Lowest-priced equivalent generic
LPGW: Lowest paid unskilled government workers
MPR: Median price ratio
MSH: Management Sciences for Health
NCD: Non-communicable diseases
NGO: Nongovernmental organizations
OB: Originator brand
PHCC: Primary Health Care Corporation
PPP: Purchasing power parity
SD: Standard deviation
UMIC: Upper-middle income country
WHO/HAI: World Health Organization and Health Action International

## Footnotes

1 Mark-up: The additional costs to the manufacturer selling price (MSP) in the form of charges and costs to cover different stages of the distribution chain including profits, overheads cost, and distribution fees. Regressive mark-up: lower mark-up for higher priced medicines.

2 Landed price is the second out of five price components. It is the price of a shipped medicine that had cleared customs and import duties , transported to wholesalers or importer. It is the cost of the procurement process.
